# Use of aromatase inhibitors to treat endometriosis-related pain symptoms: a systematic review

**DOI:** 10.1186/1477-7827-9-89

**Published:** 2011-06-21

**Authors:** Simone Ferrero, David J Gillott, Pier L Venturini, Valentino Remorgida

**Affiliations:** 1 Department of Obstetrics and Gynaecology, San Martino Hospital and University of Genoa, Italy; 2 St. Bartholomew's School of Medicine & Dentistry, Queen Mary University of London, London, UK

## Abstract

This systematic review aims to assess the efficacy of aromatase inhibitors (AIs) in treating pain symptoms caused by endometriosis. A comprehensive literature search was conducted to identify all the published studies evaluating the efficacy of type II nonsteroidal aromatase inhibitors (anastrozole and letrozole) in treating endometriosis-related pain symptoms. The MEDLINE, EMBASE, PubMed, and SCOPUS databases and the Cochrane System Reviews were searched up to October 2010. This review comprises of the results of 10 publications fitting the inclusion criteria; these studies included a total of 251 women. Five studies were prospective non-comparative, four were randomized controlled trials (RCTs) and one was a prospective patient preference trial. Seven studies examined the efficacy of AIs in improving endometriosis-related pain symptoms, whilst three RCTs investigated the use of AIs as post-operative therapy in preventing the recurrence of pain symptoms after surgery for endometriosis. All the observational studies demonstrated that AIs combined with either progestogens or oral contraceptive pill reduce the severity of pain symptoms and improve quality of life. One patient preference study demonstrated that letrozole combined with norethisterone acetate is more effective in reducing pain and deep dyspareunia than norethisterone acetate alone. However, letrozole causes a higher incidence of adverse effects and does not improve patients' satisfaction or influence recurrence of symptoms after discontinuation of treatment. A RCT showed that combining letrozole with norethisterone acetate causes a lower incidence of adverse effects and lower discontinuation rate than combining letrozole with triptorelin. Two RCTs demonstrated that, after surgical treatment of endometriosis, the administration of AIs combined with gonadotropin releasing hormone analogue for 6 months reduces the risk of endometriosis recurrence when compared with gonadotropin releasing hormone analogue alone. In conclusion, AIs effectively reduce the severity of endometriosis-related pain symptoms. Since endometriosis is a chronic disease, future investigations should clarify whether the long-term administration of AIs is superior to currently available endocrine therapies in terms of improvement of pain, adverse effects and patient satisfaction.

## Background

Endometriosis is a chronic estrogen dependent gynaecological condition characterized by the presence of ectopic glands and stroma outside the uterine cavity. It affects at least 3.6% of women [1] and it often causes infertility and/or pain symptoms (dysmenorrhea, deep dyspareunia, chronic pelvic pain and dyschezia). In some patients, pain symptoms are extremely severe and negatively affect quality of life, work efficiency and sexual life [2-4]. Several hormonal therapies have been proposed for the treatment of endometriosis related pain, including oral contraceptive pill and other estroprogestin formulations (such as the vaginal ring and the transdermal patch), progestins (such as medroxyprogesterone acetate, norethisterone acetate and the levonorgestrel-releasing intrauterine device), gonadotrophin releasing hormone analogues and danazol [5]. These traditional endocrine therapies for endometriosis inhibit estrogens production in the ovary. However, in some patients, pain symptoms may persist despite the use of endocrine therapies.

Since the late 1990s, several independent studies based either on polymerase chain reaction or immunohistochemistry have demonstrated that aromatase P450 is over-expressed in both eutopic and ectopic endometrium of patients with endometriosis, while this enzyme is not detectable in eutopic endometrium obtained from healthy women and in endometriosis free peritoneal tissue [6-13]. Although the aromatase P450 can been detected in both epithelial and stromal cells obtained from ectopic and eutopic endometrium of women with endometriosis, its expression is higher in epithelial than in stromal cells. In contrast with majority of the literature, some recent studies questioned the aberrant expression of the aromatase P450 in endometriosis [14-16]. Delvoux *et al*. reported the absence of aromatase P450 in eutopic and ectopic tissue obtained from women with endometriosis [14]. In addition, it was demonstrated that endometriotic lesions could create a hyperestrogenic environment increasing the reduction of estrone into 17-β estradiol and decreasing the oxidation of 17-β estradiol into estrone [14]. In agreement with these observations, Colette *et al*. found that aromatase P450 is undetectable by immunohistochemistry in the glandular and stromal compartments of ectopic endometrial tissue [16]. Furthermore, the authors showed that the expression of the aromatase gene, measured by quantitative polymerase chain reaction using three different protocols, is low in endometriomas and barely detectable in only a small percentage of eutopic endometrial samples, peritoneal lesions and rectovaginal nodules [16]. The authors suggested that what was believed to be aromatase protein was mainly endogenous biotic labeling or iron deposits [15].

Based on the molecular observations of increased expression of aromatase P450 in endometriotic tissue published over the last ten years, some authors used aromatase inhibitors (AIs) to treat pain symptoms in premenopausal women with endometriosis [17,18]. This systematic review was performed to assess the efficacy of AIs in treating pain symptoms caused by endometriosis.

## Methods

This systematic review was carried out according to the MOOSE guidelines [19]. No institutional review board approval was required because only published, de-identified data were analysed.

### Identification of the literature

A systematic literature search was performed to identify all the published studies evaluating the efficacy of AIs in treating pain symptoms associated with endometriosis. The search included the combination of the following medical subjects heading terms: "endometriosis", "deep endometriosis", "deeply infiltrating endometriosis", "rectovaginal endometriosis", "ovarian endometriosis", "endometriomas", "bowel endometriosis", "bladder endometriosis", "dysmenorrhea", "pelvic pain", "deep dyspareunia", "medical therapy/treatment", "aromatase inhibitors", "letrozole" and "anastrozole".

The following electronic databases were searched MEDLINE, EMBASE, PubMed, SCOPUS and the Cochrane System Reviews from inception until October 2010.

All pertinent articles were examined and their reference lists were systematically reviewed in order to identify other studies for potential inclusion in this systematic review. Review articles, books and monographs published on endometriosis were consulted and their reference lists were systematically searched to identify further studies that could be reviewed.

No language restriction was applied. The search was run every month between January 2009 and October 2010 to identify new articles.

### Study selection

Randomised controlled trials (RCTs), patient preference trials and observational studies were included in this review, whereas case reports, abstracts and proceedings of scientific meetings were excluded.

The studies included in the current review were selected accordingly to the following criteria:

- Population. Premenopausal women with diagnosis of endometriosis (either as a primary or recurrent disease) based at least on vaginal and/or rectal examination; ideally on previous surgery and histological examination of the lesions. Patients complaining of pain symptoms such as dysmenorrhea, deep dyspareunia, chronic pelvic pain and dyschezia.

- Interventions. Treatment of pain symptoms with third generation nonsteroidal (type II) AIs either alone or in combination with other hormonal therapies.

- Design. The minimum number of individuals in each trial was 10. The minimum size of follow-up was 80% or more, though there was no minimum length of follow-up. Assessment of the intensity of pain symptoms by using standardised scales such as the visual analogue scale (VAS).

- Outcome. Changes in the intensity of endometriosis-related pain symptoms during treatment with AIs either alone or combined with other hormonal therapies but not combined with surgery (primary outcome). Efficacy of AIs either alone or combined with other hormonal therapies in preventing the recurrence of pain after surgical treatment of endometriosis (secondary outcome).

The abstract of studies retrieved in the search were reviewed by two authors (S.F. and D.J.G.) to exclude citations deemed irrelevant. The reviewers worked independently and in duplicate. Any discrepancy between the two reviewers was resolved by consensus or arbitration by a third reviewer (V.R.). The reviewers were not blinded to the names of investigators or sources of publication.

### Data extraction

Data were extracted from each article and collected in standardised forms in duplicate by two reviewers (S.F. and D.J.G.). A final abstraction form was compiled from the two evaluation forms, with correction and resolution of any discrepancy between reviewers by consensus reached after discussion or arbitration by a fourth reviewer (P.L.V.).

## Results

### Study identification

**Figure 1:**
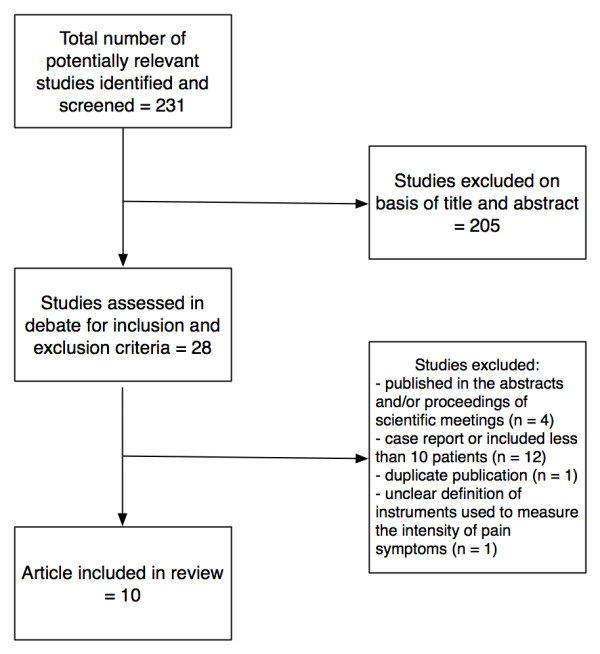
**Study selection flow chart**.

Figure 1 shows the flow diagram of the literature search results. The search identified 231 articles of which 28 abstracts reported findings on the treatment of endometriosis with aromatase inhibitors. These articles were retrieved for detailed assessment. Of the 28 studies found, 4 were excluded because they were only published in the abstracts or proceedings of scientific meetings [20-23], 12 were excluded because they were case reports or included less than 10 patients [24-35], 1 study was excluded because of duplicate publication [36] and 1 study was excluded because the instruments used to describe the changes in pain symptoms during treatment were not clearly defined [37].

### Characteristics of the included studies

Data on the efficacy of AIs in treating endometriosis-related pain symptoms were extracted from the remaining 10 articles, all of which were published in full in peer-reviewed journals between 2004 and October 2010 [38-47]. Of the ten included studies, five were prospective non-comparative [38,39,41-43], four were RCTs [39,45-47] and one was a prospective patient preference trial [44]. Four studies were performed in Italy [42-44,47] two in USA [38,40], two in Iran [45,46], one in Turkey [39] and one in Austria [41]. A total of 251 women with endometriosis treated with aromatase inhibitors were found, of which 62 were in prospective non-comparative studies, 148 were in RCTs and 41 in the patient-preference trial. A total of 183 patients were treated with letrozole and 68 patients were treated with anastrozole. The AI was administered vaginally in one study [41], in all the other studies it was administered orally. Three studies investigated the use of AIs as post-operative therapy in preventing the recurrence of pain after surgical treatment of endometriosis [39,45,46]; seven studies examined the effect of AIs in improving endometriosis-related pain symptoms. The characteristics of the studies included in the review are described in Table 1.

**Table 1:** Characteristics of the study included in the systematic review

Authors	Year	Study design	Number of subjects	Age of the patients (years)	Diagnostic modality	Characteristics of endometriosis	Intervention: aromatase inhibitor	Intervention: other hormonal therapies	Intervention: non-hormonal therapies	Post-operative treatment	Length of treatment	Criteria for pain evaluation
Ailawadi et al. [38]	2004	Prospective non-comparative trial	10	29.9 (22-45)^a^	histology	ASRM stage^b^: stage 1, n = 1 stage 2, n = 1 stage 3, n = 3 stage 4, n = 6	oral: letrozole 2.5 mg/day	oral: norethisterone acetate 2.5 mg/day	oral: elemental calcium 1250 mg/day, vitamin D 800 I.U./day	no	6 months	VAS
Soysal et al. [39]	2004	Randomised controlled trial	40	31.3 ± 5.7^b^	surgery	ASRM stage^b^: stage 4, n = 40	oral: anastrozole 1 mg/day	subcutaneous: goserelin 3.6 mg/4 weeks	oral: elemental calcium 1200 mg/day, vitamin D 800 I.U./day	yes	6 months	TPSS
Amsterdam et al. [40]	2005	Prospective non-comparative trial	18	23-46^c^	surgery	NA	oral: anastrozole 1 mg/day	oral: ethinyl estradiol 20 μg/day, levonorgestrel 0.1 mg/day	none	no	6 months	VAS
Hefler et al. [41]	2005	Prospective non-comparative trial	10	31.2 ± 4.3^b^	histology	rectovaginal endometriosis	vaginal: anastrozole 0.25 mg/day	none	oral: elemental calcium 1200 mg/day, vitamin D 800 I.U./day	no	6 months	VAS
Remorgida et al. [42]	2007	Prospective non-comparative trial	12	32.8 ± 3.2^b^	surgery, US, MRI	ASRM stage^b^: stage 4, n = 12	oral: letrozole 2.5 mg/day	oral: desogestrel 75 μg/day	oral: elemental calcium 1000 mg/day, vitamin D 880 I.U./day	no	6 months	VAS
Remorgida et al. [43]	2007	Prospective non-comparative trial	12	32.3 ± 3.8^b^	histology, US, MRI	rectovaginal endometriosis	oral: letrozole 2.5 mg/day	oral: norethisterone acetate 2.5 mg/day	oral: elemental calcium 1000 mg/day, vitamin D 880 I.U./day	no	6 months	VAS
Ferrero et al. [44]	2009	Prospective patient preference trial	41	31.2 ± 4.6^b^	histology, US	rectovaginal endometriosis	oral: letrozole 2.5 mg/day	oral: norethisterone acetate 2.5 mg/day	oral: elemental calcium 1000 mg/day, vitamin D 880 I.U./day	no	6 months	VAS
Roghaei et al. [45]	2010	Randomised controlled trial	38	32.3 ± 6.0^b^	surgery	NA	oral: letrozole 2.5 mg/day	none	oral: elemental calcium 1000 mg/day, vitamin D 880 I.U./day	yes	6 months	11-item scale
Alborzi et al. [46]	in press	Randomised controlled trial	47	29.2 ± 5.3^b^	histology	ASRM stage^b^: stage 1-2, n = 24 stage 3-4, n = 23	oral: letrozole 2.5 mg/day	none	none	yes	2 months	VAS
Ferrero et al. [47]	in press	Randomised controlled trial	35	35.1 ± 3.8^b^	surgery, US	rectovaginal endometriosis	oral: letrozole 2.5 mg/day	oral norethisterone acetate 2.5 mg/day (n = 17) or intramuscular triptorelin 11.25 mg/3 months (n = 18)	oral: elemental calcium 1000 mg/day, vitamin D 880 I.U./day	no	6 months	VAS and VRS

^a ^mean, range;^b ^mean ± SD;^c ^range

US = ultrasonography; MRI = magnetic resonance imaging; ASRM = American Society for Reproductive Medicine stage of endometriosis [51]; VAS = visual analogue scale; TPSS = total pelvic symptom score; VRS = verbal rating scale; NA = not available

### Changes in the intensity of endometriosis-related pain symptoms during treatment with AIs (primary outcome)

Seven studies included in this review investigated the efficacy of AIs in treating endometriosis-related pain symptoms (Table 2).

**Table 2:** Main results of the studies investigating the efficacy of AIs in improving endometriosis-related pain symptoms

Authors	Year		Pain at baseline (mean ± SD)	Pain et the end of treatment (mean ± SD)	Completion of treatment (%, n)	Quality of life during treatment (instrument)	Satisfaction with treatment (%,n)	Lesion size at baseline (cm^3^)	Lesion size at the end of treatment (cm^3^)
Ailawadi et al. [38]	2004	pain score	6.22 ± 2.07	2.52 ± 2.09	100% (10/10)	N.A.	N.A.	N.A.	N.A.
Amsterdam et al. [40]	2005	pain score	8.70 ± 1.76	3.20 ± 2.70	83.3% (15/18)	N.A.	N.A.	N.A.	N.A.
Hefler et al. [41]	2005	dysmenorrhea chronic pelvic pain	3.6 ± 1.9 1.9 ± 1.4	3.1 (1.6) 1.9 (1.3)	100% (10/10)	Improved (SF-36)	N.A.	4.2 (median)	4.2 (median)
Remorgida et al. [42]	2007	dysmenorrhea deep dyspareunia chronic pelvic pain	8.7 ± 1.9 6.5 ± 2.7 6.0 ± 1.9	0.8 ± 0.7 0.6 ± 0.5 3.2 ± 2.6	0% (0/12)	N.A.	N.A.	N.A.	N.A.
Remorgida et al. [43]	2007	dysmenorrhea deep dyspareunia chronic pelvic pain	8.8 ± 1.0 7.6 ± 1.5 5.6 ± 0.9	3.7 ± 2.2 2.2 ± 2.0 2.4 ± 1.6	100% (10/10)	Improved (SF-36)	N.A.	N.A.	N.A.
Ferrero et al. [44]	2009	deep dyspareunia chronic pelvic pain	6.6 ± 2.1 5.9 ± 1.6	1.7 ± 1.1 1.5 ± 1.4	90.2% (37/41)	N.A.	56.1% (23/41)	N.A.	N.A.
Ferrero et al. [47]	in press	*Triptorelin group* deep dyspareunia chronic pelvic pain *Norethisterone acetate *group deep dyspareunia chronic pelvic pain	6.4 ± 1.9 6.1 ± 1.4 6.6 ± 2.1 6.0 ± 1.4	2.0 ± 0.9 1.2 ± 1.3 2.2 ± 1.4 2.0 ± 1.8	55.6% (10/18) 94.1% (16/17)	N.A. N.A.	22.2% (4/18) 64.7% (11/17)	3.2 (mean) 3.4 (mean)	2.8 (mean) 3.0 (mean)

#### Prospective non-comparative studies

In an open-label, phase 2, nonrandomised prospective study, Ailawadi *et al*. examined the efficacy of letrozole in treating reproductive age women with endometriosis and chronic pelvic pain [38]. The study included ten women with endometriosis-related pain symptoms that persisted after previous surgical and medical treatment. In particular, each patient had at least one course of treatment with leuprolide acetate for 3-6 months before enrolment in the study. Each patient underwent a diagnostic laparoscopy to histologically confirm the diagnosis of endometriosis and establish the ASRM stage of the disease. No ablation of endometriotic lesions was performed. Letrozole (2.5 mg/day) and norethisterone acetate were administered within 1 month from laparoscopy and continued for 6 months. During the first month of treatment, pain score improved in 9 patients (90.0%). At the completion of the 6-month treatment, there was a significant decrease in the intensity of pain symptoms. Only one patient (10.0%) had persistent pain during treatment. In addition, a second laparoscopy performed within 2 months of the completion of therapy demonstrated that the treatment improved the ASRM stage of endometriosis.

In another open-label, phase 2, non-randomised prospective study, Amsterdam *et al*. treated with anastrazole (1 mg/day) and oral contraceptive pill 18 women with endometriosis and chronic pelvic pain refractory to multiple medical and surgical treatments [40]. 15 women (83.3%) completed the 6-month treatment. Three patients interrupted the therapy because of persistence of pain in one case, anxiety in another case and loss to follow-up in another case. Improvements in pain symptoms were observed at 1 month after commencing the treatment. At the completion of the study, a significant reduction in the intensity of pelvic pain was reported in 14 of 15 patients (93.3%).

Hefler *et al*. administered anastrozole (0.25 mg/day) in a vaginal suppository to 10 women with histologically confirmed rectovaginal endometriosis [41]. After 6 months of treatment, an improvement in dysmenorrhea was observed in all but one patient; however, the baseline intensity of dysmenorrhea in the study population was unusually low (3.6 cm on the VAS scale) for a population of patients with rectovaginal endometriosis. The intensity of chronic pelvic pain and dyspareunia was unchanged by treatment, as was the number of days with the use of analgesic. The therapy resulted in a significant improvement in quality of life (measured by the Short Form-36). Data on the changes in the volume of the rectovaginal endometriotic nodules during the six-month treatment were reported in 9 women; the volume decreased in three patients, remained stable in three and increased in three. Three patients (30%) underwent surgery after the completion of treatment.

More recently, in an open-label prospective study, Remorgida *et al*. administered letrozole (2.5 mg/day) and desogestrel to 12 women with endometriosis-related pain symptoms that were refractory to previous medical and surgical treatments [42]. All the patients interrupted the treatment after a median time of 84 days (range, 56-112 days) because of the development of functional ovarian cysts and 8 women (66.7%) developed more than one ovarian cyst. At the interruption of treatment, the intensity of dyspareunia was significantly decreased when compared with baseline values. In contrast, no statistically significant change was observed in the reported intensity of chronic pelvic pain. Pain symptoms recurred after the discontinuation of treatment and at 6-month follow-up their intensity was similar to baseline values.

In a further open-label prospective study, Remorgida *et al*. administered letrozole (2.5 mg/day) and norethisterone acetate to 12 women with histologically confirmed rectovaginal endometriosis [43]. The intensity of deep dyspareunia and chronic pelvic pain dropped significantly after only 1 month of treatment and continued to decrease during therapy. After 6-month treatment, the intensity of chronic pelvic pain and deep dyspareunia were significantly lower than at baseline; in addition, there was a significant improvement in quality of life (measured by the Short Form-36). Pain symptoms recurred after discontinuation of treatment and, after 6 months from the completion of treatment there was no significant difference in the intensity of pain symptoms when compared with pre-treatment values. Five patients (41.7%) underwent surgery at 7.0 (± 2.5) months after the completion of therapy.

#### Prospective patient-preference study

In a prospective, open-label, patient preference study including 82 women with rectovaginal endometriosis, Ferrero *et al*. compared the efficacy and tolerability of letrozole (2.5 mg/day) combined with norethisterone acetate with norethisterone acetate alone [44]. At the completion of treatment, no significant difference was observed in patient satisfaction between the two study groups; 63.4% of the patients receiving norethisterone acetate alone and 56.1% of those receiving the double-drug regimen were either satisfied or very satisfied with the treatment. At both 3 and 6 months of treatment, the intensity of chronic pelvic pain and deep dyspareunia were significantly decreased when compared with baseline values in the two study groups. In line with this observation, the use of analgesic medications significantly decreased during the treatment in both study groups. However, at both 3-and 6-month assessment, the intensity of chronic pelvic pain and deep dyspareunia were significantly lower in women receiving the double-drug regimen than in those receiving norethisterone acetate alone. Pain symptoms quickly recurred after the discontinuation of treatment without significant differences between the two study groups. Adverse effects of treatment were significantly more frequent in patients treated with the double drug-regimen (43.2%) than in those receiving norethisterone acetate alone (18.4%; p = 0.020). The authors concluded that the combination drug regimen was more effective in reducing pain and deep dyspareunia than norethisterone acetate alone. However, letrozole caused a higher incidence of adverse effects and did not improve patient satisfaction or delay recurrence of symptoms after discontinuation of treatment.

#### Randomised controlled study

In a RCT, Ferrero *et al*. assigned symptomatic women with rectovaginal endometriosis treated with letrozole (2.5 mg/day) to receive either norethisterone acetate or triptorelin for 6 months [47]. The study was ended pre-term based on the results of an interim analysis, which was performed when 35 women had been recruited. The interim analysis showed that a significantly higher number of patients interrupted the treatment because of adverse effects when letrozole was combined with triptorelin (44.4%; 8/18) than when it was combined with norethisterone acetate (5.9%, 1/17; p = 0.018). In line with this finding, the percentage of women satisfied with treatment was significantly higher in those treated with norethisterone acetate (64.7%) than in those treated with triptorelin (22.2%; p = 0.028). The intensity of both chronic pelvic pain and deep dyspareunia significantly decreased during treatment in both study groups, though no meaningful difference between the two groups was apparent. After 6 months of treatment, there was a significant reduction in the volume of the rectovaginal nodules in both study groups; however, the reduction in the volume of endometriotic nodules was significantly greater in patients receiving triptorelin (16.1%) than in those receiving norethisterone acetate (10.2%).

### Prevention of symptom recurrence after surgery for endometriosis (secondary outcome)

Three RCTs examined whether the postoperative administration of AIs reduces the risk of endometriosis recurrence after surgery for endometriosis [39,45,46] (Table 3). Soysal *et al*. randomised 80 women who underwent radical surgical treatment of severe endometriosis to receive either goserelin and anastrozole or goserelin alone for 6 months [39]. All the patients included in the RCT received elemental calcium and vitamin D. The RCT found that the double-drug regimen reduced the proportion of women experiencing recurrence of pain at 24 months' follow-up compared with goserelin alone (8% with combination treatment versus 35% with goserelin alone). Furthermore, combination treatment significantly increased the median time to symptom recurrence compared with goserelin alone (greater than 2.4 months with combination treatment versus 1.7 months with goserelin alone; RR 4.3, 95% CI 1.3 to 9.8; p = 0.0089). At the completion of treatment the menopausal quality of life (measured by the modified Greene scale and the Blatt-Kupperman Index) was not statistically significant different between the two study groups.

**Table 3:** Main results of the three RCT investigating the efficacy of AIs in preventing symptoms recurrence after surgery for endometriosis (secondary outcome)

Authors	Treatment	Length of treatment	Length of follow-up after discontinuation of hormonal therapy	Results
Soysal et al. [39]	- anastrozole (1 mg/day) or placebo - goserelin 3.6 mg/4 weeks - elemental calcium (1200 mg/day), vitamin D (800 I.U./day)	6 months	24 months	The recurrence rate was 7.5% (3/40) in patients receiving anastrozole and goserelin and 35.0% (14/40) in patients receiving goserelin alone. The median time to detect symptom recurrence was > 24 months in patients receiving anastrozole and goserelin and 17 months in patients receiving goserelin alone.
Roghaei et al. [45]	- letrozole 2.5 mg/day or danazol (600 mg/day) or placebo - elemental calcium (1000 mg/day), vitamin D (880 I.U./day)	6 months	0 months	At the end of the treatment, the intensity of pain symptoms was significantly lower in patients treated with letrozole or danazol than in those treated with placebo.
Alborzi et al. [46]	letrozole (2.5 mg/day) or triptorelin (3.75 mg/4 weeks) or no treatment	2 months	12 months	The rate or recurrence was 6.4% (3/47) in patients treated with letrozole, 5.0% (2/40) in patients treated with triptorelin and 5.3% (3/57) in patients receiving no treatment (not significant).

In another RCT, Roghaei *et al*. randomised 106 women with endometriosis who underwent cauterization of the lesions to receive one of the following treatments: letrozole (2.5 mg/day; n = 38), danazol (600 mg/day; n = 37) or placebo (n = 31) for 6 months [45]. All the patients included in the RCT also received elemental calcium and vitamin D. The study showed that, during the treatment, the intensity of dyspareunia, dysmenorrhea and chronic pelvic pain decreased in each study group. However, at the end of the treatment, the intensity of pain symptoms was significantly lower in patients treated with letrozole or danazol than in those treated with placebo. Unfortunately, no follow-up was reported after the discontinuation of the hormonal therapy.

Alborzi *et al*. randomised 144 infertile women who underwent laparoscopic excision of endometriotic lesions to be treated with letrozole (2.5 mg/day), triptorelin (3.75 mg/every 4 weeks) or placebo for 2 months [46]. The rate of recurrence of endometriosis was similar in the three groups. At one year after the restoration of the menstrual cycle, recurrence of endometriosis was observed in 6.4% of the patients treated with letrozole, 5.0% of those treated with triptorelin and 5.3% of those receiving placebo (not significant). In addition, no significant difference was observed in the pregnancy rates of women included in the three study groups (23.4% in patients treated with letrozole, 27.5% in those treated with triptorelin and 28.1% in those receiving placebo).

## Discussion

All the observational studies included in this review [38,40-43], one patient-preference trial [44] and one RCT [47] demonstrated that AIs combined with either progestogens, oral contraceptive pill or gonadotropin releasing hormone analogue reduce the intensity of pain symptoms caused by endometriosis (Table 2). In addition, two observational studies showed that the administration of AIs improves quality of life [41,43]. The effect of AIs on the volume of rectovaginal endometriotic nodules remains unclear. In one observational non-comparative study [41], the administration of vaginal anastrozole (0.25 mg/day) for 6 months did not cause a decrease in the volume of rectovaginal endometriotic nodules. In contrast, a RCT [47] showed that the oral administration of letrozole (2.5 mg/day) combined with either gonadotropin releasing hormone analogue or progestogen significantly reduces the size of rectovaginal endometriotic nodules.

Endometriosis is a chronic disease [48] and pain typically recurs to a degree similar to that at baseline after discontinuation of endocrine therapies [49,50]. Most of the published observational non-comparative studies did not report a follow-up after discontinuation of treatment with AIs [38,40,41,47]. Two observational non-comparative studies [42,43] and one patients-preference trial [44] observed a quick recurrence of pain symptoms after discontinuation of therapy with AIs. In particular, after 6 months from the completion of the treatment, the intensity of pain symptoms was similar to pre-treatment values [42-44]. Furthermore, patients with persistent pain symptoms during the administration of AIs have been found to have rectovaginal nodules with preserved glandular epithelium and abundant stromal cells having proliferative activity [43].

The adverse effects caused by AIs in premenopausal women (such as arthralgia and myalgia) may be more severe than those caused by progestogens and oral contraceptive pill thus reducing the compliance of the patients in the long-term treatment. Recently, a patient preference study demonstrated that letrozole and norethisterone acetate are more effective in reducing pain and deep dyspareunia than norethisterone acetate; although the AI resulted in a higher incidence of adverse effects [44]. As a consequence patients' satisfaction was not improved by the administration of letrozole combined with norethisterone acetate when compared with norethisterone acetate alone [44]. In this perspective, it is preferable to combine AIs with progestogens rather than with gonadotropin releasing hormone analogues. In fact a RCT showed that combining the AI with norethisterone acetate causes a lower incidence of adverse effects and lower discontinuation rate than combining letrozole with triptorelin [47].

AIs may have a role in the prevention of recurrence after surgical treatment of endometriosis. Two RCTs [39,45] demonstrated that the administration of AIs (alone or combined with gonadotropin releasing hormone analogue) for 6 months reduces the risk of endometriosis recurrence when compared with postoperative treatment with gonadotropin releasing hormone analogue or danazol. In contrast, one RCT demonstrated that the postoperative administration of AIs for only 2 months does not decrease the risk of endometriosis recurrence when compared with gonadotropin releasing hormone analogue or placebo [46].

The heterogeneity of the analyzed studies is an important limitation of the current systematic review. In fact, AIs were administered either alone or in combination with various hormonal therapies (progestin, oral contraceptive pill or gonadotropin releasing hormone analogues). In addition, the severity of endometriosis was established accordingly to the classification of the American Society for Reproductive Medicine (ASRM) only in four studies [51]; furthermore, two of these studies included women with various stage of disease. Four studies included patients with rectovaginal endometriosis, but the severity of the disease was not established accordingly to the ASRM classification. A further limitation of this review consists in the fact that AIs were administered only for a relatively short period of time (from two to six months). Despite these important limitations, the findings of the analysed studies suggest the efficacy of AIs in the short-term treatment of endometriosis-related pain symptoms. However, women with endometriosis should receive a chronic treatment [5,48] and the optimal therapy should balance potential benefits and side affects. In this perspective, the role of AIs in the long-term treatment of pain symptoms caused by endometriosis remains uncertain because of the persistence of endometriotic lesions during treatment [43] and of the high incidence of the side effects observed not only in women with endometriosis [44,47] but also in premenopausal breast cancer patients [52]. In addition, there are several concerns on the safety of a long-term administration of AIs to premenopausal women. Third-generation AIs negatively affect bone health [53,54]. They suppress aromatase activity within the osteoblasts presumably favouring increased osteoclastic activity and net loss of bone mineral density [53]. One RCT included in this review [47] and studies performed in premenopausal breast cancer patients [55] showed that a 6 month treatment with AIs and gonadotropin releasing hormone analogue significantly decrease bone mineral density. The hypoestrogenism caused by AIs may be associated with other adverse effects. Studies performed in breast cancer patients showed that AIs may lack the lipid lowering and cardioprotective effect of tamoxifen, but they do not seem to increase the risk of cardiovascular events [56]. However, data on the effects of AIs on cardiovascular disease is limited, particularly in premenopausal women.

Given this background, it seems unlikely that AIs, particularly combined with gonadotropin releasing hormone analogues, may be used as a standard long-term treatment of premenopausal endometriosis. Combining AIs with other hormonal therapies (such as norethisterone acetate) may decrease the incidence of adverse affects and reduce the loss of bone mineral density. However, only women failing to improve after the administration of standard endocrine therapies and surgical excision of endometriosis may be candidate to receive AIs. These agents should be prescribed only to women with histologically proven endometriosis and normal mineral bone density. In addition, before starting the treatment, patients should be informed in details of the potential adverse effects caused by AIs, particularly musculoskeletal symptoms.

## Conclusions

In conclusion, this systematic review suggests that AIs effectively reduce the severity of endometriosis-related pain symptoms. Since endometriosis is a chronic disease, future investigations should clarify whether the long-term administration of AIs is superior to currently available endocrine therapies in terms of improvement of pain, adverse effects and patient satisfaction. The potential risks caused by the hypoestrogenism associated with the administration of AIs (particularly on bone mineral density) should always be considered before prescribing these agents to premenopausal women. On the basis of the available data, aromatase inhibitors should now be offered only to women who have severe pain despite previous surgical and hormonal therapies [18].

## Competing interests

The authors declare that they have no competing interests.

## Authors' contributions

All the authors contributed to the conception of the review. SF and DJG performed literature search, selected the abstracts and abstracted the data. VR resolved the dicrepancies between the two reviewers (SF and DJG) in the selection of the study of interest. PLV resolved the dicrepancies between the two reviewers (SF and DJG) in the abstraction of the data from the study of interest. SF prepared the first draft of the manuscript and performed subsequent amendments. PLV and VR reviewed the manuscript. All authors read and approved the final manuscript.
